# The Application of Novel Drug Delivery Systems in the Treatment of Osteoarthritis

**DOI:** 10.3390/pharmaceutics17101272

**Published:** 2025-09-29

**Authors:** Pengfei Huang, Junjie Zhao, Xiyu Wang, Zhaokun Zhang, Haiyan Zhao

**Affiliations:** 1 The First Clinical College of Medicine, Lanzhou University, Lanzhou 730000, China; huangpf2023@lzu.edu.cn (P.H.); zhaojj2023@lzu.edu.cn (J.Z.); wxiyu2023@lzu.edu.cn (X.W.); zhzhaokun2023@lzu.edu.cn (Z.Z.); 2 Department of Orthopedics, The First Hospital of Lanzhou University, Lanzhou 730000, China

**Keywords:** delivery system, osteoarthritis, nanoparticles

## Abstract

Osteoarthritis (OA) is a degenerative joint disease characterized by cartilage degradation, synovial inflammation, and abnormal bone remodeling. Current therapies, such as NSAIDs, corticosteroids, and hyaluronic acid injections, primarily alleviate symptoms but often cause systemic side effects and fail to modify disease progression. Novel drug delivery systems (NDDS), including liposomes, polymer microspheres, nanoparticles, hydrogels, and biomimetic carriers, have emerged to enhance drug targeting, prolong retention, and reduce toxicity. These systems enable controlled release of anti-inflammatory, antioxidant, and gene therapies, improving therapeutic outcomes. However, challenges remain in biocompatibility, scalability, and clinical translation. Future efforts should focus on optimizing material design, functionality, and personalized approaches to facilitate the clinical application of NDDS for OA treatment.

## 1. Introduction

OA is a chronic degenerative joint disorder marked by the deterioration of articular cartilage, inflammation of the synovial membrane, and alterations in the structure of the subchondral bone. This condition predominantly impacts middle-aged and elderly individuals and is recognized as a significant contributor to functional impairment and a reduction in quality of life among older adults globally [1]. As the global population ages at an accelerated rate, the prevalence and associated burden of OA are on the rise, imposing significant challenges for both individual patients and public health infrastructures [2].

The pathological mechanisms underlying OA are intricate and encompass various pathological alterations, including cartilage degeneration, inflammatory responses, oxidative stress, and modifications in subchondral bone. The principal characteristic of OA is the progressive deterioration of articular cartilage. Matrix metalloproteinases (MMPs) and enzymes from the ADAMTS (a disintegrin and metalloproteinase with thrombospondin motifs) family are pivotal in the degradation of cartilage matrix proteins, such as type II collagen and aggrecan. Notably, MMP-13 and ADAMTS-5 are recognized as critical contributors to the pathogenesis of OA [3]. Furthermore, persistent inflammation of the synovial membrane within the joint cavity serves as a significant contributor to the advancement of OA. Pro-inflammatory mediators, including interleukin-1 beta (IL-1β) and tumor necrosis factor-alpha (TNF-α), facilitate the upregulation of MMPs and a disintegrin and ADAMTS through the activation of the nuclear factor kappa-light-chain-enhancer of activated B cells (NF-κB) and mitogen-activated protein kinase (MAPK) signaling pathways. This process ultimately exacerbates cartilage degradation and intensifies the inflammatory response [4]. Throughout the onset and progression of OA, the overproduction of reactive oxygen species (ROS) contributes to chondrocyte apoptosis and the degradation of the extracellular matrix. This process significantly exacerbates the advancement of OA by impairing the mitochondrial function of chondrocytes and triggering inflammatory pathways [5]. OA not only impacts cartilage but also contributes to the calcification of subchondral bone, the development of osteophytes, and the reduction in joint space. Specifically, atypical mechanical stresses and inflammatory factors within the bone matrix collectively induce a disruption in the process of bone remodeling, thereby intensifying the lesions associated with OA [6].

At present, the therapeutic approach to OA primarily emphasizes the alleviation of symptoms and the mitigation of disease progression. However, there is a notable deficiency in disease-modifying treatments capable of repairing joint architecture or reversing the trajectory of the disease [7]. For example, OA treatment currently emphasizes symptom management, utilizing commonly employed interventions such as NSAIDs, intra-articular injections of hyaluronic acid, and corticosteroids [8]. However, prolonged use of these therapies may result in adverse effects, including gastrointestinal damage, cardiovascular complications, and chondrotoxicity. Furthermore, these approaches primarily serve to alleviate pain and inflammation without addressing the underlying degeneration of cartilage or impeding the disease’s progression [9]. The efficacy of drug delivery in the intra-articular environment is notably limited due to the high fluidity of synovial fluid, which facilitates the rapid clearance of administered medications. Additionally, the dense matrix of cartilage presents a significant barrier to drug penetration, complicating the effective targeting of therapeutic agents to the relevant cellular populations [10]. In addition, the long-term administration of NSAIDs is associated with gastrointestinal and cardiovascular side effects, while intra-articular corticosteroid injections may induce chondrocyte toxicity and structural damage to the joint. The challenge of individualized treatment is further exacerbated by the considerable variability in the pathological mechanisms and severity of OA among patients, coupled with the lack of specificity in existing treatment modalities [11]. In conclusion, OA is a multifaceted degenerative condition characterized by complex pathological mechanisms, including inflammation, oxidative stress, and tissue degeneration. Current therapeutic strategies predominantly focus on symptom relief, with a notable deficiency in effective interventions aimed at reversing disease progression. Consequently, the advancement of innovative drug delivery systems designed to enhance the targeting and stability of therapeutic agents represents a critical avenue for future research, offering potential new avenues for disease-modifying treatments in OA.

**Figure 1:**
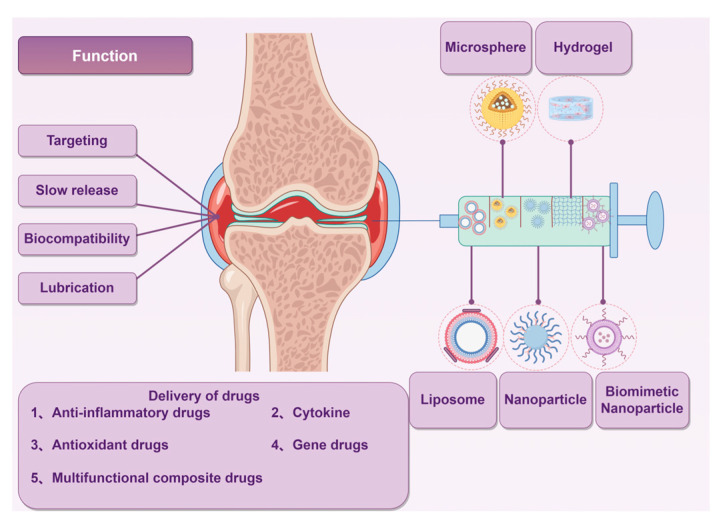
The mechanism of NDDS in the treatment of osteoarthritis (by Figdraw 2.0).

The Drug Delivery System (DDS) has achieved notable advancements in the management of OA through enhancements in drug stability, targeting efficacy, and sustained release capabilities. Recent investigations indicate that novel drug delivery systems (NDDSs) can effectively address the challenges of rapid drug clearance and inadequate targeting associated with conventional treatment approaches. For instance, liposomes facilitate prolonged sustained release by encapsulating NSAIDs, thereby minimizing systemic toxicity. Empirical evidence suggests that these systems significantly ameliorate synovial inflammation and mitigate cartilage degeneration [12]. Polymer nanoparticles, through surface modification, augment the capacity of pharmaceuticals to traverse the cartilage matrix, thereby markedly enhancing the delivery efficiency of anti-inflammatory agents and drugs aimed at cartilage repair [13]. Hydrogels, recognized as an innovative class of drug delivery systems, possess a three-dimensional network architecture that facilitates the adsorption of pharmaceuticals for prolonged release. Furthermore, these hydrogels are capable of delivering drugs in a targeted manner, responding to specific microenvironmental conditions, such as pH levels or enzymatic activity at sites of inflammation. This targeted release mechanism contributes to minimizing drug wastage and mitigating potential side effects [14,15]. Moreover, biomimetic delivery systems, exemplified by cell membrane-coated nanoparticles, replicate inherent biological properties, resulting in prolonged drug retention and precise delivery within the joint cavity, thereby augmenting therapeutic efficacy [16,17]. Recent reviews on NDDS for OA have primarily focused on traditional systems like liposomes and nanoparticles. In contrast, this review emphasizes three underdiscussed advancements: 1. Multifunctional NDDS combining anti-inflammatory, antioxidant, and regenerative therapies. 2. Application progress of NDDS, including liposomal dexamethasone and hydrogel-based siRNA delivery, etc. 3. Structural engineering breakthroughs, such as zwitterionic polymer brushes for joint lubrication and ROS-scavenging material networks. By integrating these themes, we offer a roadmap for overcoming translational barriers in OA therapy, as presented in Figure 1.

## 2. The Basic Principles of Novel Drug Delivery Systems (NDDS)

**Figure 2:**
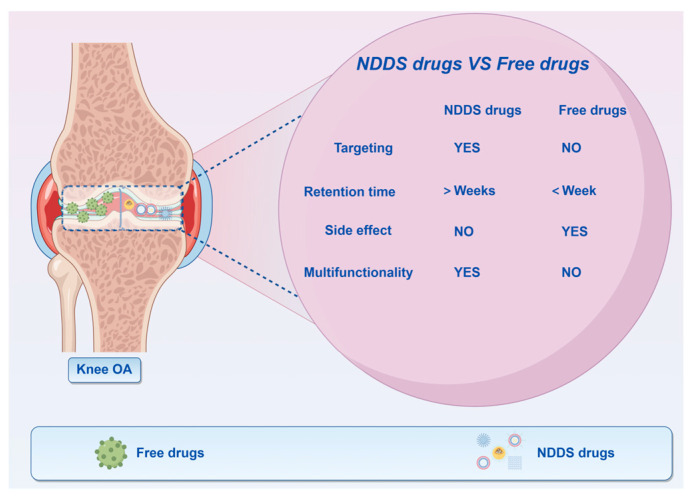
Comparison of the advantages of NDDS drugs and free drugs in the treatment of osteoarthritis (by Figdraw).

Novel drug delivery systems (NDDS) for osteoarthritis (OA) address the shortcomings of conventional therapies by employing four fundamental mechanisms: targeted delivery, sustained release, biocompatibility, and multifunctionality. This section provides a comprehensive analysis of the structural and functional attributes of prominent NDDS, integrating recent research developments, and illustrates their comparative efficacy relative to traditional pharmacological treatments, as depicted in Figure 2.

### 2.1. Material Structure and Targeting Mechanism

#### 2.1.1. Liposome

Liposomes are spherical vesicles formed by lipid bilayers, with the membrane material primarily consisting of natural lipid molecules such as phospholipids and cholesterol. Their particle size ranges from 1 nm to 10 μm. In this structure, hydrophilic drugs can be encapsulated in the aqueous environment inside the liposome, while lipophilic drugs can be dispersed within the lipid bilayer [18]. Modifications to the administration technique, delivery site, and particle size of liposomes can facilitate targeted delivery. Furthermore, the incorporation of specific recognition molecules onto the liposomal structure enables selective targeting through their affinity for particular target cells [19].

#### 2.1.2. Polymer Microspheres

Microspheres are defined as spherical particles characterized by a diameter within the micrometer range. In the domain of drug delivery, polymer microspheres are diminutive spheres or sphere-like entities created by the dissolution or dispersion of a pharmaceutical agent within polymeric materials, generally exhibiting a particle size between 1 and 250 μm. The mechanism of drug loading in microspheres entails the physical embedding or adsorption of the drug onto the surface or within the polymer matrix, with the inherent stability of the polymer facilitating a controlled and sustained release of the drug [20]. The incorporation of specific targeting ligands, including antibodies, receptors, or glycosyl groups, onto the surface of microspheres enables these particles to selectively identify and adhere to corresponding receptors or ligands present on target cells or tissues. This process facilitates the achievement of active targeting [21].

#### 2.1.3. Nanoparticles

Nanoparticles are defined as solid colloidal entities with dimensions typically spanning from 1 to 1000 nanometers. Pharmaceutical compounds can be either dissolved or encapsulated within polymeric matrices to create drug-loaded nanoparticles [22]. The surfaces of nanoparticles can be engineered with particular ligands or antibodies that possess the ability to identify and attach to receptors present on target cells. Through the interaction with these specific ligands, nanoparticles can be preferentially internalized by the intended cells, which subsequently increases the localized concentration of the therapeutic agent at the desired site [23].

#### 2.1.4. Hydrogel

A hydrogel is defined as a gel in which water acts as the dispersing medium. It is comprised of water-soluble polymers that exhibit a cross-linked network structure, which includes both hydrophobic groups and hydrophilic residues. The hydrophilic residues interact with water molecules, facilitating their incorporation into the network, whereas the hydrophobic residues undergo swelling in the presence of water, thereby contributing to the formation of the cross-linked polymer structure [24]. Hydrogels can be engineered to deliver drugs, bioactive molecules, or other therapeutic agents with precision to designated tissues, organs, or cellular locations by employing surface modification techniques, targeted ligands, and specific biomolecule recognition. This approach not only improves therapeutic efficacy but also minimizes adverse effects on healthy tissue [25].

#### 2.1.5. Bionic Delivery Materials

Bionic delivery materials represent a novel advancement in drug delivery systems, aimed at harnessing endogenous natural biomaterials to create nanoscale carriers for targeted drug delivery via the body’s intrinsic pathways. The principal emphasis is on cell membrane bionic delivery systems, which are predominantly sourced from mammalian cells and their derivatives, including red blood cells, platelets, white blood cells, immune cells, and extracellular vesicles. By capitalizing on the inherent characteristics of these cells and their derivatives, such as the ability to evade immune responses and achieve homologous targeting, it is possible to facilitate precise drug delivery [26]. For instance, biomimetic nanocarriers derived from red blood cell membranes have the capability to prolong the circulation duration of pharmaceuticals within the organism. In contrast, biomimetic materials derived from platelet membranes possess the ability to specifically target injured blood vessels and neoplastic cells. Specifically, nanoparticles that mimic red blood cell membranes can replicate the strong affinity exhibited by surface molecules on red blood cells for collagen, thereby facilitating the accurate targeting of trabecular meshwork tissues that are abundant in extracellular matrix components [27].

### 2.2. Treatment Mechanism

The fundamental concept underlying NDDS is to enhance the therapeutic efficacy of pharmaceuticals by means of targeted delivery, sustained release, and biocompatibility, particularly in the context of OA treatment. This approach seeks to mitigate issues such as rapid drug clearance, inadequate targeting, and limited duration of effectiveness. Primarily, NDDS facilitates the precise delivery of therapeutic agents to the affected regions by employing specific molecules, such as hyaluronic acid and cartilage-targeting peptides, or utilizing materials like liposomes and nanoparticles. This strategy aims to achieve selective drug enrichment, thereby minimizing systemic adverse effects [28]. Furthermore, NDDS enhances the duration of pharmacological effects by employing a sustained release mechanism. The utilization of polymer microspheres and hydrogels facilitates a continuous release of medication, thereby prolonging the retention time within the joint cavity and minimizing the frequency of administration [29]. In addition, NDDS enhances the biological stability of pharmaceuticals by encapsulating and safeguarding them, which subsequently diminishes their degradation within the organism [30]. Contemporary NDDS incorporate multifunctional capabilities, including the administration of anti-inflammatory medications or gene therapies via nanoparticles. Additionally, these systems can provide antioxidant or lubricating properties, thereby facilitating a synergistic approach to the treatment of OA [31]. As can be seen from Figure 2, these NDDSs can significantly improve the bioavailability and efficacy of drugs, providing new strategies for the treatment of OA.

## 3. Novel Drug Delivery Systems for OA Treatment

In this section, we will discuss the application of NDDSs (Novel Drug Delivery Systems) for the intra-articular (IA) route of administering different classes of drugs for osteoarthritis. Table 1 summarizes the schemes and efficacy of each delivery system in OA treatment and describes in detail the benefits, difficulties, and future trends for every sort of NDDS for the treatment of OA.

### 3.1. Liposome Delivery System

#### 3.1.1. Overview of Liposome Delivery Systems

Liposomes are spherical vesicles characterized by a lipid bilayer, which demonstrates biocompatibility akin to that of natural cell membranes and exhibits enhanced drug delivery capabilities. This structural configuration enables liposomes to transport both hydrophilic pharmaceuticals, which are encapsulated within the aqueous core, and hydrophobic pharmaceuticals, which are integrated into the bilayer, thereby rendering them effective carriers for drug delivery. For the most part, liposomes are composed of natural components like phospholipids or cholesterol. The combination of their tunable structures and biocompatibility reduces cell degradation in a short span of time in the human body, resulting in a prolonged duration of therapeutic activity of the entrapped drugs. Due to their biocompatibility, liposomes have been widely employed in drug delivery to treat a variety of diseases, such as cancer and infectious diseases, and OA [32]. Furthermore, liposomes’ lipid bilayer allows for sustained drug release at the target site, which helps reduce the systemic side effects of the drug. This feature is particularly important for diseases that are chronic and require long-term treatment, one of which would be OA [33].

#### 3.1.2. Progress in the Application of Liposomes in the Treatment of OA

In recent years, liposome delivery systems have gained significant traction in the management of OA. These systems primarily facilitate the administration of anti-inflammatory agents, gene therapies, and antioxidants, in addition to the advancement of multifunctional liposomes.

##### Anti-Inflammatory Drug Delivery

Low-grade inflammation is a key contributor to the pathogenesis of OA, and anti-inflammatory medications, including NSAIDs and glucocorticoids, are commonly used to treat the condition but also come with substantial systemic side effects. The use of liposome delivery systems may significantly reduce the toxic side effects of these pharmaceutical agents while ensuring long-term release in the joint cavity. He et al. [34] prepared cationic liposomes loaded together with clonixin (Lnxc-CL) using a film dispersion method and loaded microRNA-140 (miR-140) via electrostatic interactions. Their findings indicated that these liposomes not only prolonged the retention time of the drug within the joint cavity and demonstrated low cytotoxicity but also safeguarded miRNA from degradation by nucleases. This resulted in a significant reduction in synovial inflammation in a rat model of OA. In a comparable study, Teng et al. [35] employed liposomes as a delivery system for dexamethasone, which resulted in the mitigation of OA and pain symptoms in mice deficient in miR-204/-211. This therapeutic effect was attributed to the facilitation of synovial macrophage repolarization towards the M2 phenotype. Furthermore, Liu et al. [36] achieved a notable enhancement in the sustained release of the drug at the OA site by modifying liposomes with polyethylene glycol (PEG), thereby improving the drug’s efficacy.

##### Cytokine Delivery

Transforming growth factor beta (TGF-β) is integral to the initial development of articular cartilage, the preservation of homeostasis, and the facilitation of cartilage repair. Nevertheless, the efficacy of traditional injection methods is hindered by the short half-life of growth factors and their vulnerability to proteolytic degradation, which complicates the maintenance of stable cytokine concentrations within the body. Typically, achieving sustained release and physiological effects necessitates the administration of high doses. In contrast, liposome delivery systems offer a mechanism for continuous release. Research conducted by Jiang et al. [37] demonstrated that encapsulating TGF-β1 within liposomes allows for prolonged delivery while sustaining effective therapeutic concentrations in the cellular microenvironment. Additional studies have indicated that liposomal carriers can enhance the stability of anti-inflammatory cytokines, such as IL-10, and prolong the retention time of therapeutic agents [38].

##### Antioxidant Delivery

Oxidative stress is a significant factor in the pathophysiology of OA, contributing to the susceptibility of synovial and chondrocyte cells to damage. Antioxidants, such as astaxanthin and glycyrrhizin, have been shown to mitigate the levels of ROS within the joint cavity through the use of liposome delivery systems. Zhao et al. [39] developed a liposomal formulation containing astaxanthin, which demonstrated efficacy in eliminating excess ROS and nitric oxide (NO) in the osteoarthritic microenvironment. This reduction in oxidative stress subsequently diminished the infiltration of synovial macrophages and alleviated associated inflammation and tissue damage. In addition, these effects of liposomal nanoparticles on cartilage tissue repair provide new insights into the mechanism of action of astaxanthin on OA. Another study developed a liposomal carrier of liquiritin that had ROS scavenging ability to alleviate the degradation of cartilage matrix and slow the progression of OA [40].

##### Gene Drug Delivery

Gene therapy has emerged as a new modality for OA therapeutics; however, gene-based therapeutics are prone to instability via degradation in the biological milieu. As a potential solution to this problem, the use of liposomes for gene delivery as a carrier has been investigated to protect the therapeutic activity of the drugs. In a study conducted by Wang et al. [41], small interfering RNA (siRNA) was encapsulated within cationic lipid complexes using electrostatic interactions with positively charged liposomes. The findings indicated that siRNA encapsulated in liposomes demonstrated resistance to enzymatic degradation and immune system challenges while also enhancing the efficiency of endocytosis. This approach effectively inhibited the expression of pro-inflammatory genes within the joint cavity, contributing to the mitigation of cartilage damage and the postponement of OA progression.

Furthermore, liposome carriers have been employed for the delivery of microRNA (miRNA). Xue et al. [42] developed a bisphosphonate (BP) liposome-like material characterized by a strong affinity for bone minerals. This innovative carrier demonstrates the capability to effectively transport miRNA therapeutic agents to the bone microenvironment in vivo, thereby offering novel therapeutic strategies and avenues for bone regeneration.

##### Multifunctional Liposome Delivery

To enhance therapeutic efficacy, recent efforts have concentrated on the development of multifunctional liposome carriers capable of co-delivering anti-inflammatory agents, antioxidants, cartilage regeneration-promoting compounds, and targeted therapeutic agents. Li et al. [43] devised a multifunctional liposome system that encapsulates the cartilage regeneration-promoting drug Teriparatide PTH (1-34) using a film dispersion technique. The resulting PTH (1-34)-loaded liposomes (Lipo@PTH (1-34)) were subsequently integrated into a solution of gallic acid-grafted gelatin (GGA), which possesses antioxidant properties, leading to the formation of injectable hydrogels through transglutaminase (TG enzyme) crosslinking. This innovative system not only demonstrates favorable biocompatibility and non-toxicity but also facilitates the synthesis of glycosaminoglycans (GAGs), thereby protecting the cartilage layer from degeneration, mitigating the progression of OA, and promoting cartilage repair. Such multifunctional delivery systems enhance the therapeutic effects of the drugs while concurrently reducing the dosage of each component, thereby minimizing the potential for adverse effects. Furthermore, through the application of targeted modification technology, multifunctional liposomes can more precisely target diseased tissues, thereby improving drug utilization efficiency. Li et al. [44] employed microfluidic technology to incorporate liposomes modified with a cartilage-affinity peptide (WYRGRL) that carries a mitochondrial autophagy activator (Urolithin A) into hyaluronic acid methacrylate hydrogel microspheres. This delivery system is instrumental in promoting the recovery of mitochondrial function, clearing reactive oxygen species, and maintaining chondrocyte homeostasis by enhancing mitochondrial autophagy. It underscores the significant potential of mitochondria-targeted subcellular strategies in the treatment of OA.

#### 3.1.3. Summary

The liposome delivery systems have unique features such as high targeting efficiency, efficient sustained release, multifunctional drug loading, and suitable biocompatibility, hence showing great potential for OA treatment. These systems can enhance therapeutic efficiency through targeted release of drugs and reduction in systemic toxicity. In addition, High production costs (500–1000 per dose) limit scalability, but PEGylated formulations show promise in early trials. However, despite these advantages provided by liposomes for the treatment of osteoarthritis, there are still some limitations: 1. Stability Concerns in Vivo: Liposomes can be degraded by serum proteins and enzymes in the body, potentially limiting the prolonged activity of the encapsulated drug. The rapid degradation of liposomes may lead to uncontrollable release of drugs, negatively affecting the therapeutic efficacy. 2. Complex Process: The preparation of liposomes requires complex techniques and specialized equipment, which makes stability control challenging and leads to variability between production batches and higher manufacturing costs. 3. Limited Drug Loading Capacity: The small internal volume of liposomes restricts their capacity for drug carriage, which could be particularly problematic for patients suffering from severe stages of osteoarthritis requiring substantial drug quantities, as the drug loading capacity of one liposomal dose may be insufficient for its continuous use in producing therapeutic effects.

These three limitations hinder the broader application of liposome systems. Future research should prioritize enhancing the in vivo stability of liposomes, increasing their drug loading capacity, and bolstering the accumulation of clinical data to ensure that liposomal formulations can achieve optimal efficacy and safety in the treatment of OA.

### 3.2. Polymer Microsphere Delivery System

#### 3.2.1. Overview of Polymer Microsphere Delivery System

The polymer microsphere delivery system is a biocompatible, polymer-based, micron-sized drug delivery carrier with a sustained-release and targeting function. Materials often used in the process include polylactic acid (PLA), polylactic acid-co-glycolic acid (PLGA), and chitosan. These polymers can also degrade by a controlled process within the body and release encapsulated drugs or bioactive molecules. Most polymer microspheres have a particle size between 1 and 100 microns, which can be retained in tissues for an extended period of time, for example, in joint cavities, and achieve sustained release of drugs [45]. Through manipulation of polymer type/molecular weight/preparation techniques, scientists are able to generate a range of microsphere architectures that deliver highly specific, controlled drug release therapeutic requirements.

#### 3.2.2. Progress in the Application of Polymer Microspheres in the Treatment of OA

The polymer microsphere delivery system serves as a micron-sized vehicle for drug administration. Current research on polymer microspheres in the context of osteoarthritis treatment predominantly emphasizes the use of NSAIDs, glucocorticoids, and anti-inflammatory cytokines. Additionally, significant advancements have been made in the areas of antioxidants and gene therapy.

##### NSAIDs Delivery

NSAIDs are frequently utilized in the management of OA due to their efficacy in alleviating pain and inflammation. However, traditional oral or injectable NSAIDs may lead to adverse effects, particularly gastrointestinal discomfort. To mitigate systemic side effects, polymer microsphere systems facilitate localized drug delivery. In a study conducted by Yurtdaş-Kırımlıoğlu et al. [46], a polyphosphazene-based ibuprofen microsphere system was developed through the synthesis of novel cross-linked inorganic hybrid polyphosphazene microspheres via self-assembly precipitation polymerization. The in vitro characterization of these microspheres revealed their spherical morphology and micron size, along with a prolonged release profile, indicating their potential to enhance analgesic and anti-inflammatory effects. Additionally, another investigation employed chitosan microspheres for the delivery of diclofenac sodium. Findings from this study demonstrated that in a rabbit model of arthritis, the anti-inflammatory and sustained-release properties of compound hydrogel microspheres containing diclofenac sodium surpassed those of both the drug solution and pure chitosan hydrogel. Consequently, these compound hydrogel microspheres present a promising drug delivery system that may improve the therapeutic efficacy of diclofenac sodium, thereby providing a significant preliminary foundation for the development of novel intra-articular drug delivery systems [47].

##### Glucocorticoid Delivery

Glucocorticoids, such as dexamethasone, are recognized for their efficacy in alleviating joint inflammation; however, their systemic administration can lead to adverse effects, including immunosuppression. To mitigate systemic toxicity while ensuring localized and sustained drug release, researchers have employed polymer microsphere carriers for the delivery of dexamethasone. Roach et al. [48] investigated dexamethasone-loaded PLGA microspheres, which are designed to facilitate prolonged drug release within the joint cavity, thereby minimizing the detrimental effects associated with local pro-inflammatory cytokine exposure and safeguarding articular cartilage. Additionally, Fang et al. [49] utilized hyaluronic acid-sodium alginate microspheres for the encapsulation of dexamethasone, which not only prolongs the drug’s half-life in the organism and decreases the frequency of injections but also demonstrates remarkable protective effects on cartilage while significantly alleviating inflammation and pain in a rat model of OA.

##### Delivery of Anti-Inflammatory Cytokines

The utilization of anti-inflammatory cytokines, including Interleukin-4 (IL-4), Interleukin-10 (IL-10), and Interleukin-13 (IL-13), has been shown to mitigate inflammation while simultaneously promoting protective metabolic responses in chondrocytes [50]. Empirical evidence supports the notion that these cytokines inhibit the secretion of matrix metalloproteinases, the degradation of proteoglycans, and the apoptosis of chondrocytes [51]. In essence, anti-inflammatory cytokines possess both anti-catabolic and anabolic properties that contribute to the preservation of cartilage integrity. However, conventional cytokine injections are often subject to rapid metabolic degradation, which complicates the maintenance of effective therapeutic concentrations. In contrast, polymer microspheres offer a mechanism for sustained release of cytokines. Research conducted by Park et al. [52] has demonstrated the development of bioresponsive microspheres utilizing a gelatin solution within an oil-in-water emulsion, wherein the anti-inflammatory cytokines IL-4, IL-10, and IL-13 are cross-linked through electrostatic adsorption. Findings indicate that these bioresponsive gelatin microspheres not only prolong the half-life of the anti-inflammatory cytokines but also diminish their clearance during phases of reduced disease activity. Furthermore, microspheres containing IL-4 and IL-13, when co-cultured with osteoarthritis chondrocytes, have been shown to reduce inflammation by as much as 80%. This on-demand delivery system, which is synchronized with catabolic responses, holds significant potential for applications in wound healing, particularly in the prevention of inflammation-mediated cartilage damage associated with OA.

##### Antioxidant Delivery

Oxidative stress is a critical factor in the pathophysiology of OA, with ROS contributing to the deterioration of cartilage and synovial cells. The utilization of polymer microsphere carriers has been shown to effectively deliver antioxidants, such as arbutin and tannic acid, to targeted areas, thereby mitigating oxidative stress. In a study conducted by Jin et al. [53], gelatin methacryloyl liposomes (GM-Lipo@ARB) were developed to encapsulate arbutin (ARB), demonstrating sustained release of ARB and notable cartilage-targeting capabilities. The ARB-loaded microspheres significantly attenuated the inflammatory response in arthritis chondrocytes exposed to interleukin (IL-1β). Moreover, the synthesized GM-Lipo@ARB microspheres exhibited anti-inflammatory properties by inhibiting NF-κB signaling and exerted antioxidant effects through the activation of the Nrf2 pathway, which in turn regulated extracellular matrix (ECM) homeostasis in chondrocytes and mitigated the progression of OA in murine models. Additionally, another investigation introduced innovative porous injectable nanofiber microspheres loaded with tannic acid (TA), which interacted with adjacent hydroxyl groups in TA and strontium (Sr) metal ions to form a three-dimensional stable metal-phenolic network (MPN). These microspheres not only demonstrated remarkable bioactivity, structural integrity, antioxidant capabilities, and favorable cell compatibility but also exhibited enhanced scavenging activity against reactive oxygen species, as well as the capacity to inhibit cartilage degradation and stimulate the secretion of cartilage-specific ECM [54].

##### Gene Drug Delivery

Gene therapy represents a novel approach for the treatment of OA, with the capacity to modulate joint inflammation and safeguard cartilage integrity. RNA interference (RNAi), a technique aimed at influencing cellular outcomes at the messenger RNA (mRNA) level, has emerged as a pivotal strategy in the field of tissue regeneration [55]. RNAi encompasses molecules such as microRNA (miRNA) and small interfering RNA (siRNA); however, these molecules often exhibit rapid dispersion and lack specificity in cellular targeting when administered systemically, typically resulting in transient knockdown effects lasting less than one week [56,57]. Consequently, the sustained and localized delivery of RNAi molecules is recognized as a critical therapeutic avenue for further exploration. Polymer microspheres have been identified as effective vehicles for the stable delivery of gene therapeutics to the joint cavity, thereby mitigating gene degradation and enhancing transfection efficiency. Sun et al. [58] demonstrated the transfer of miR-24 mimics encapsulated within PLGA microspheres to Skeletal Muscle Satellite Cell (SMSC) organoids, which facilitated intracellular delivery, improved in vivo stability, and enabled targeted delivery of the miR-24 mimics. Moreover, PLGA microspheres offer the advantage of prolonging the circulation half-life of miRNA, thereby allowing for sustained and controlled release. The PLGA microspheres containing miR-24 mimics were subsequently formulated into an aging-targeted miR-24 microsphere/SMSC organoid composite hydrogel (MSOH). Research findings indicate that the MSOH hydrogel effectively repairs cartilage defects within the OA microenvironment by enhancing the miR-24/TAOK1 signaling pathway, suggesting its potential as a novel therapeutic option for cartilage repair in patients with osteoarthritis-related bone defects. In addition, another investigation developed light-crosslinked dextran microspheres (MS) that encapsulated siRNA-micelles via an aqueous emulsion technique, which were then integrated into human Mesenchymal Stem Cell (hMSC) aggregates for localized and sustained delivery of bioactive siRNA. This study revealed that the microspheres released siRNA micelles continuously over a 28-day period, with the released siRNA maintaining its capacity to transfect cells for gene silencing, thereby underscoring its significant potential for applications in tissue regeneration [59].

#### 3.2.3. Summary

Recently, due to the advantages of polymer microsphere systems, such as sustained release, targeted delivery, and good biocompatibility, polymer microsphere systems have been widely applied in the management of OA. Such systems can achieve effective stabilization of local drug concentrations, with low systemic side effects. Examples include NSAIDs, corticosteroids, cytokines, antioxidants, and gene therapies, all of which can be incorporated into polymer microspheres to provide significant anti-inflammatory and antioxidant responses, along with cartilage protection. However, there is still room for improvement in other aspects, like degradation control, biotoxicity, and preparation methods. Further research shall focus on optimizing the degradation characteristics of microsphere materials, the ability to target more tissues more effectively, and preparing techniques to make clinical usage of polymer microspheres in treating OA more broadly in the future.

### 3.3. Nanoparticle Delivery System

#### 3.3.1. Overview of Nanoparticle Delivery Systems

Nanoparticle delivery systems are nanoscale materials that can act as drug carriers, with particle sizes ranging from 10 to 1000 nanometers. These systems, with their microsize, can cross biological barriers and enter the cells, making them very effective in the treatment of various diseases, ranging from cancer and inflammatory disorders to OA. Commonly used materials for the construction of these systems include poly(lactic-co-glycolic acid) (PLGA), chitosan, and silica, which exhibit good biocompatibility and biodegradability. Additionally, the surfaces of nanoparticles can be functionalized with antibodies, ligands, or biomolecules to enhance their targeting capability and biodistribution [60,61].

#### 3.3.2. Progress in the Application of Nanoparticles in OA Treatment

In recent years, investigations into nanoparticle-mediated drug delivery systems for the treatment of OA have predominantly concentrated on anti-inflammatory therapies, gene therapy, antioxidant applications, and the regeneration of cartilage. The subsequent discussion highlights significant research developments that have emerged over the past five years.

##### Delivery of Anti-Inflammatory Drugs

Non-steroidal anti-inflammatory drugs (NSAIDs) and glucocorticoids are frequently used to treat OA to reduce inflammation, yet they induce adverse systemic effects, including gastrointestinal distress and immunosuppression. There is accumulating evidence showing that the selective delivery of anti-inflammatory treatment to the joint cavity is possible through nanoparticle systems that provide sustained anti-inflammatory effects while reducing systemic toxicity. Kang et al. [62] thermoresponsiveF127/COS/KGNDCF nanoparticles that can rapidly release anti-inflammatory drugs, like diclofenac, as well as conduct sustained release of cartilage regenerative factors. The liberation of both drugs in such a manner has shown promising results in the reduction of inflammation and promotion of cartilage healing. Moreover, Whitmire et al. [4] described the development of self-assembling nanoparticles that transport anti-inflammatory proteins like interleukin-1 receptor antagonists (IL-1Ra) directly to the joint cavity and extend their time retention within the organism. This novel system increases the bioavailability of anti-inflammatory proteins and decreases the systemic adverse effects at the same time.

##### Delivery of Antioxidants

Oxidative stress exacerbates the degeneration of chondrocytes and the occurrence of synovitis. Research has demonstrated that antioxidants can effectively mitigate oxidative stress-related damage to the joints. In their study, Luo et al. [5] examined the antioxidant capabilities of metal oxide nanoparticles, including manganese dioxide and cerium oxide, which function by mimicking the activity of antioxidant enzymes to diminish reactive oxygen species, thereby decelerating the progression of OA. Furthermore, Zhong et al. [63] developed dopamine-melanin nanoparticles that exhibit broad-spectrum antioxidant properties, capable of scavenging excess reactive oxygen and nitrogen species while enhancing autophagy activity, thus offering substantial protection against cartilage degeneration in OA models.

##### Gene Drug Delivery

Small interfering RNA (siRNA) and microRNA (miRNA) have the capacity to suppress the expression of pro-inflammatory genes through mechanisms of gene silencing, thereby mitigating joint inflammation. In their research, Bedingfield et al. [3] developed nanoparticles that deliver siRNA specifically targeting matrix metalloproteinase 13 (MMP13). This approach effectively diminished MMP13 expression by focusing on type II collagen, which in turn helped to preserve the structural integrity of articular cartilage and decelerated the advancement of OA. Additionally, a review conducted by Householder et al. [64] examined the utilization of gene therapy in the management of OA, encompassing gene editing technologies and RNA delivery systems aimed at repairing damaged cartilage and postponing the progression of the disease.

##### Cartilage Repair and Regeneration

A collagen II-targeted nanoparticle platform represents an advanced precision drug delivery system that has been developed through the application of nanotechnology, with the primary objective of facilitating targeted therapy for lesions associated with collagen II in various diseases. Collagen II serves as the principal structural protein within cartilage tissue, comprising the majority of cartilage matrix proteins, and is predominantly found in both articular and hyaline cartilage. Its significance is particularly pronounced in the context of osteoarthritic conditions, including osteoarthritis and rheumatoid arthritis. Xue et al. [65] designed a metal–organic framework (MOF)-based nanoparticle platform that enhances autophagy and provides protection to cartilage by enabling the combined delivery of rapamycin and bilirubin, thereby effectively delaying the degeneration of cartilage in OA. Furthermore, nanoparticles modified with cartilage-targeting peptides constitute a sophisticated precision drug delivery platform that utilizes nanotechnology to achieve targeted delivery of therapeutic agents or biomolecules for the treatment of cartilage-related disorders, such as OA, rheumatoid arthritis, and cartilage injuries. This technology also holds a great promise to enhance therapeutic efficacy, reduce side effects, and provide a unique platform for precision medicine in cartilage diseases via the presence of peptide targeting for a cartilage entity on the surface of these nanoparticles. Rothenfluh et al. [66] examined nanoparticles formed with the cartilage-targeting peptide WYRGRL. The drug delivery efficiency was improved through collagen II-targeted release, and the retention time in the joint was prolonged, promoting cartilage repair.

Adult stem cells, particularly mesenchymal stem cells (MSCs), have shown immense promise for cartilage regeneration and tissue repair; however, the survival of MSCs directly injected into the joint cavity is severely limited. Nanoparticles can be utilized as carriers or recruitment facilitators, providing a protective medium for stem cells, thereby improving their ability to survive and differentiate in the joint microenvironment. Another interesting study demonstrated the facilitation of the spatial delivery of cells using a new technology by labeling MSCs in vitro with superparamagnetic iron oxide (SPIO) particles [67]. A subsequent magnetic force is used to guide MSCs to the target region of cartilage defects. SPIO-labeled MSCs were specifically targeted to cartilage defects by a magnetic field, leading to a higher local concentration of MSCs. In addition to this, these cells successfully generated a three-dimensional multilayer type “sheet” structure without the utilization of any scaffold or membrane support.

#### 3.3.3. Summary

Nanoparticle-based drug delivery systems have received significant interest in the treatment of osteoarthritis as they are highly efficient at penetrating cells and can effectively deliver gene therapies and anti-inflammatory drugs on account of the small size and high surface area offered by nanoparticles, which occupy a large volume relative to their mass. These systems also enjoy high targeting accuracy, high sustained-release ability, multi-functionality, and improved cellular uptake efficiency. Although nanoparticle systems have shown significant potency in treating osteoarthritis, they do suffer from some limitations related to their in vivo metabolism, long-term safety, as well as scalability for manufacture. Future studies need to focus on the optimization of nanoparticle materials to ameliorate their biocompatibility and degradability while reinforcing clinical trials to promote their practical application in OA therapy.

### 3.4. Hydrogel Delivery Systems

#### 3.4.1. Overview of Hydrogel Delivery Systems

Hydrogels are a type of polymeric material with a three-dimensional network structure that can hold large amounts of water and become soft and rubbery. Hydrogel materials have a very wide application in drug delivery, tissue engineering, and regenerative medicine due to their high water content, mechanical properties similar to soft tissue, and great biocompatibility. Hydrogels are mostly composed of natural and synthetic polymers [68,69] that can create durable 3D architectures, such as chemical or physical cross-linking. The structural rigidity enables sustained drug release after the loading of the therapeutic agents [70]. Under OA conditions, hydrogels can maintain longer treatment effects through localized drug delivery and stabilizing the joint residence time [71].

#### 3.4.2. Progress in the Application of Hydrogels in OA Treatment

Investigations into the utilization of hydrogels for the treatment of osteoarthritis have predominantly concentrated on the formulation of anti-inflammatory agents, antioxidants, gene therapies, stem cell applications, and multifunctional delivery systems. The subsequent section outlines significant progress made in this field over the past five years.

##### Hydrogel Delivery of Anti-Inflammatory Drugs

The systemic administration of conventional anti-inflammatory drugs, including NSAIDs and glucocorticoids, may result in adverse effects such as gastrointestinal discomfort. To address this issue, hydrogel delivery systems have been developed to facilitate the sustained release of anti-inflammatory agents directly into the joint cavity, thereby minimizing systemic toxicity. Wang et al. [72] introduced a single-component hydrogel formulated from polycitric acid ester, which demonstrates the ability to scavenge ROS in vivo, modulate the inflammatory microenvironment, and significantly reduce the expression of the cartilage-degrading enzyme MMP-13, thus providing protection to articular cartilage. Furthermore, Zhang et al. [73] created a thermosensitive hydrogel encapsulating glucosamine (GlcN), which effectively diminishes joint swelling and the expression of inflammatory mediators by prolonging drug release, thereby offering a promising approach for the treatment of OA.

##### Hydrogel Delivery of Antioxidants

Oxidative stress is a significant mechanism involved in the pathophysiology of OA, where an overproduction of ROS can exacerbate damage to synovial cells and chondrocytes. In response to this challenge, researchers have employed hydrogels as a means to deliver antioxidants, thereby mitigating oxidative stress within the joint cavity. Valentino et al. [74] developed an injectable system by combining chitosan nanoparticles infused with hydroxytyrosol and a thermosensitive hydrogel to alleviate inflammatory oxidative stress and prevent cartilage degeneration. Additionally, Shi et al. [75] engineered a dynamically cross-linked hyaluronic acid hydrogel endowed with ROS-scavenging properties, which serves to protect embedded chondrocytes from injury induced by the inflammatory microenvironment.

##### Hydrogel Delivery of Gene Drugs

Through the application of small interfering RNA (siRNA) and microRNA (miRNA), which can mitigate inflammatory responses by suppressing the expression of pro-inflammatory genes within the joint cavity. Nonetheless, gene therapeutics are prone to rapid degradation within the biological environment, and the use of hydrogels has been shown to significantly enhance the stability of these gene drugs. Yu et al. [76] developed a hydrogel that mimics the extracellular matrix for the delivery of genetically modified adipose-derived stem cells, resulting in improved anti-inflammatory and cartilage-protective effects while simultaneously reducing joint degeneration. Similarly, Garcia et al. [77] encapsulated anti-ADAMTS5 gapmers within fibrin-hyaluronic acid hydrogels, facilitating sustained release and effectively inhibiting the expression of genes associated with cartilage degeneration.

##### Hydrogel Delivery of Stem Cells and Growth Factors

Stem cells and growth factors are critical components in the repair of cartilage affected by OA. However, the direct injection of stem cells into the joint cavity poses challenges, including susceptibility to mechanical injury and immune rejection, which can hinder their viability. The use of hydrogels offers a protective microenvironment that enhances the survival and directed differentiation of stem cells within the joint cavity. Bhattacharjee et al. [78] explored the application of amniotic membrane hydrogel combined with adipose-derived Ktrt stem cell sequence and found that this method produced a strong anti-inflammatory and cartilage protective effect, which was beneficial for the repair of osteoarthritis joints. Furthermore, Li et al. [79] utilized integration of stem cells, antioxidant epigallocatechin gallate (EGCG), and hyaluronic acid in one hydrogel carrier that protected stem cells that were injured by inflammation and ROS.

##### Multifunctional Hydrogel Delivery Systems

Over the past few years, researchers have made considerable progress in the design of multi-functional hydrogel systems for the simultaneous delivery of various bioactive factors. Dong et al. [80] developed a double-crosslinked hydrogel with anti-inflammatory, antioxidant, and cartilage regeneration effects, significantly improving the repair of cartilage in the body. Similarly, Han et al. [81] devised hydrogel microspheres illustrating superlubricity and drug release abilities, which significantly enhanced OA therapeutic effects.

#### 3.4.3. Summary

Owing to good biocompatibility, large sustained release capabilities, multi-functional composite delivery, and controllable triggering, hydrogel delivery systems are the best candidate for drug administration and tissue regeneration. These composites lead to on-site delivery of anti-inflammatory agents, antioxidants, gene therapies, and growth factors, resulting in prolonged treatment and multi-target therapy, which effectively relieve osteoarthritis symptoms. Nonetheless, hydrogels exhibit several limitations concerning their mechanical properties, drug release regulation, and biodegradability. Furthermore, certain hydrogel materials may generate acidic byproducts during degradation, potentially triggering local inflammatory responses and adversely affecting patient tolerance. In addition, injectable thermosensitive hydrogels are advancing to Phase III for OA pain relief. Consequently, future research endeavors should prioritize enhancing the mechanical strength of hydrogels, refining their responsive release mechanisms, and improving their biodegradability and immunological stability to advance the clinical application of hydrogels in the treatment of OA.

### 3.5. Biomimetic Delivery Systems

#### 3.5.1. Overview of Biomimetic Delivery Systems

Innovative delivery systems, such as biomimetic delivery systems, are technological methodologies that mimic biological traits of biological entities present in nature for drug delivery. Such systems can more efficiently and safely target therapeutics to particular lesions by traditionally mimicking natural biological structures and physiological functions, thus decreasing the noticeable side effects on healthy tissues. Motivation for such systems mainly comes from, but is not limited to, various biological elements such as the cell membranes, ECM, exosomes, and cell membrane-coated nanoparticles [82]. Main biomimetic delivery systems included are listed as cell membrane-coated nanoparticles, ECM-mimicking materials, biomimetic proteins, and multifunctional hydrogels. Incorporating biomimetic technology through the above-mentioned materials could considerably increase the stability, targeting accuracy, and long-term release of the delivery systems [83].

#### 3.5.2. Progress in the Application of Biomimetic Delivery Systems in OA Treatment

In recent years, substantial advancements have been achieved in the research concerning biomimetic delivery systems for OA. This progress primarily encompasses the development of cell membrane-coated nanoparticles, exosome biomimetic systems, and ECM biomimetic materials.

##### Cell Membrane-Coated Nanoparticles

Cell membrane-coated nanoparticles, such as those derived from red blood cells, macrophages, and exosomes, which have high biocompatibility and immune escape ability, improve drug delivery by extending circulation time and enhancing targeting in the body. Yu et al. [84] developed a biomimetic nanoparticle (NM-LANPs@Ru) employing neutrophil membranes for the early diagnosis and treatment of OA. The new delivery system can effectively deliver by encapsulating polyethylene in centrosomal microtubules and is able to target inflammatory regions during neutrophil infiltration. Neutrophil Membrane Coated Glycol-Modified L-Arginine Nanoparticles (LANPs). In vitro and in vivo studies showed that NM-LANPs@Ru exhibited a prominent anti-inflammatory effect, significantly inhibiting OA-associated inflammation, as well as chondrocyte apoptosis, thus providing a potential strategy for the early therapeutic intervention of OA. Moreover, red blood cell membrane-camouflaged nanoparticles of various types have been investigated to enable specific drug release in inflamed environments owing to their innate properties for immune evasion. The circulation time of such nanoparticles in vivo is increased due to the natural immunoregulatory functions of the red blood cell membrane. Moreover, these surface-modified NCs can deliver anti-inflammatory agents, including NSAIDs, directly to the inflamed joint. In vivo studies indicate that this red blood cell membrane-coated system is especially favorable for reducing the adverse effects of drugs in inflammation-related diseases, including osteoarthritis (OA), while also improving the concentration and retention of therapeutic agents at the injury sites [85].

##### Exosome Biomimetic Delivery System

Exosomes are vesicles of nanoscale format that are released from cells and involved in intercellular communication by carrying many types of biomolecules, such as proteins, lipids, mRNA, and miRNA. These vesicles exhibit intrinsic targeting ability and biocompatibility. The exosome biomimetic delivery system utilizes the unique properties of exosomes to package therapeutic agents, allowing precise delivery to inflammatory cells in the joint cavity. For example, research by Liang et al. [86] involved engineering of exosomes for delivery of microRNA-140 (miR-140) to chondrocytes, and this led to a greatly decreased expression of inflammation and cartilage degradation, thus slowing up the development of OA. This targeted delivery is by targeting the chondrocyte-affinity peptide-modified exosomes. Furthermore, Zhang et al. [87] showed that exosomes from decellularized extracellular matrix-primed bone marrow mesenchymal stem cells (BMSCs) modulated the miR-3473b/PTEN pathway to exert a protective effect and promote the OA cartilage repair signaling pathway. Additionally, Wang et al. [6] found that embryonic mesenchymal stem cell-derived exosomes induce the joint tissue and, thus, mitigate the progression of OA by maintaining a balance between the synthesis and degradation of the chondrocyte ECM. In addition, exosome-based NDDS face regulatory hurdles due to standardization challenges but dominate patent filings (62% of 2023 OA NDDS patents).

##### ECM Biomimetic Materials

The ECM constitutes a three-dimensional structural network that facilitates cellular growth and tissue regeneration. Biomimetic materials that replicate the ECM exhibit significant potential for applications in osteoarthritis treatment, as they can offer sites for cell adhesion, stimulate cellular growth and differentiation, and function as systems for drug delivery. Yu et al. [76] developed an injectable hydrogel that mimics the ECM, serving as a carrier for the delivery of adipose-derived stem cells (ADSCs) and creating a conducive microenvironment for the diffusion and proliferation of these cells. In a rat model of osteoarthritis induced surgically, the intra-articular administration of hydrogels containing ADSCs resulted in a marked reduction in cartilage degeneration, joint inflammation, and subchondral bone loss, thereby enhancing anti-inflammatory and cartilage-protective effects and mitigating the progression of osteoarthritis. Additionally, Chen et al. [88] engineered biomimetic super-lubricating drug-loaded nanospheres (MSNs-NH2@PMPC-DS) through the polymerization of PMPC brushes onto the surfaces of drug nanocarriers. The PMPC polymer brush, which possesses a zwitterionic charge analogous to that of phosphatidylcholine lipids, significantly improved lubrication, while the nanocarrier MSNs facilitated sustained drug delivery by encapsulating the anti-inflammatory agent DS. Both in vitro and in vivo studies demonstrated that these biocompatible nanospheres not only shielded chondrocytes from oxidative stress-induced degradation but also inhibited the progression of osteoarthritis in a DMM animal model.

##### Biomimetic Protein Delivery System

Biomimetic protein materials, including hyaluronic acid (HA) and collagen, exhibit the capacity to engage with biological constituents within the joint cavity, thereby offering adhesion sites for pharmacological agents and facilitating sustained release in vivo. Whitmire et al. [4] devised a self-assembling protein nanoparticle system aimed at the delivery of IL-1Ra, which markedly extended the retention time of the drug and enhanced its anti-inflammatory efficacy within the joint cavity.

##### Biomimetic Delivery Systems for Gene Therapy

Gene drug delivery has the potential to modulate the inflammatory processes within the joint cavity at a molecular level, thereby mitigating the progression of OA. Delivery systems that are encapsulated by cell membranes or coated with biomimetic materials serve to protect gene drugs from degradation while enhancing targeting efficacy. Liang et al. [89] successfully delivered CRISPR/Cas9 plasmids to chondrocytes by combining exosomes and liposomes, which resulted in the specific downregulation of the MMP-13 gene and subsequently slowed the degradation of cartilage associated with OA. In a similar vein, Lu et al. [90] developed a novel non-viral gene carrier composed of hyaluronic acid and chitosan nanoparticles aimed at the targeted intracellular delivery of therapeutic genes to chondrocytes. Their findings indicated that, under identical experimental conditions, the transfection efficiency of hyaluronic acid/chitosan plasmid nanoparticles was significantly superior to that of chitosan plasmid nanoparticles alone. Furthermore, the average cell viability for hyaluronic acid/chitosan plasmid nanoparticles exceeded 90%, suggesting that the incorporation of hyaluronic acid creates a more favorable environment for the survival of chondrocytes.

#### 3.5.3. Summary

Biomimetic delivery systems, characterized by their biomimetic architecture, present several advantages, including enhanced biocompatibility, notable sustained release capabilities, and multifunctional delivery mechanisms. These attributes render them particularly effective for extended retention within the joint cavity while ensuring the maintenance of therapeutic drug concentrations. By facilitating the delivery of anti-inflammatory agents, antioxidants, gene therapies, and growth factors, these systems can significantly mitigate synovial inflammation, decelerate cartilage degeneration, and foster tissue regeneration. Nonetheless, there remains a need for further optimization and validation of biomimetic systems concerning their preparation methods, long-term safety profiles, and clinical efficacy data. Future research endeavors should prioritize the development of more efficient and safer biomimetic materials, the simplification of preparation protocols, and the execution of comprehensive clinical validations to enhance their applicability in the treatment of OA.

### 3.6. Other Delivery Systems

Micelles and lipid-based systems (solid lipid nanoparticles [SLNs], nanostructured lipid carriers [NLCs]) are emerging NDDS for OA therapy. Their amphiphilic or lipidic structures enhance drug solubility, stability, and targeted delivery.

#### 3.6.1. Micelles

Polymeric micelles self-assemble into core–shell structures, encapsulating hydrophobic drugs in the core and hydrophilic polymers in the shell. Sinani et al. [91] reviewed the design methods and current developments of polymer micelles for nucleic acid delivery, discussed the current status of polymer micelles as nucleic acid carriers, current delivery challenges, and how to improve the safety and efficacy of nucleic acids after local or systemic administration. This also provides a new approach for drug delivery for the treatment of osteoarthritis. For example, Zhou et al. [92] developed a comprehensive diagnostic and therapeutic micelle ERMs@siM13, to diagnose and intervene in early PTOA by targeting MMP13 overexpression in diseased cartilage tissue. In normal cartilage with MMP13 deficiency, ERMs@siM13 maintain a fluorescence quenching state to obtain low background noise, and due to the shielding of the cRGD ligand by PEG containing EP, non-specific cellular uptake is avoided. In contrast, after reaching diseased chondrocytes, MMP13 increases to separate PEG shells, restore Cy5 fluorescence for early OA diagnosis, and expose cRGD ligands to increase cellular internalization and on-demand treatment. Therefore, ERMs@siM13, by restoring the balance of cartilage matrix metabolism, effectively delayed the progression of early OA.

#### 3.6.2. SLNs and NLCs

Solid lipid nanoparticles (SLNs) are alternative carrier systems for liposomes, polymer nanoparticles, and inorganic carriers. In recent years, SLNs have attracted increasing attention in the delivery of drugs, nucleic acids, proteins, peptides, health products, and cosmetics. These nanocarriers have attracted attention from the industry due to their ease of preparation, physical and chemical stability, and scalability. These characteristics make SLNs an efficient and convenient drug delivery system. Ebada is the first to use cationic carriers to target anionic cartilage matrix, allowing integrated solid lipid nanoparticles (RH-SLN) to rapidly penetrate cartilage tissue and be continuously released for 3 weeks in a rat osteoarthritis model. In addition, RH-SLN significantly inhibits inflammatory response, oxidative stress, and cartilage degeneration in arthritic rats [93].

Lipid nanocarriers (NLCs) are substitutes for a class of polymer nanoparticles, liposomes, and lotion. It is mainly used for the delivery of lipophilic drugs, but its applicability to hydrophilic drugs has also been confirmed. Due to their biological non-toxicity, immunogenicity, and compatibility, NLC has become a widely studied lipid nanocarrier system [94]. In the Bhosale study, thermal homogenization was used to optimize the nanostructured lipid carriers (LRX NLCs) loaded with lornoxicam. It can be released continuously for 24 h, and the release concentration is as high as 18.9 ± 1.8% in 3 h. In addition, F14 LRX NLCs and Carbopol 940 LR gel are combined into LRX NLC gel in this study. LRX NLCs gel not only improves the pain of rats’ OA but also reduces the proliferation of synovium, inflammation, and other indicators, as well as improves the cartilage structure [95].

## 4. Challenges and Controversies

### 4.1. Biocompatibility and Safety Issues

Numerous innovative drug delivery systems have demonstrated effectiveness in preclinical animal models; however, challenges related to biocompatibility and safety continue to impede their progression to clinical application. Delivery systems such as liposomes and nanoparticles may elicit inflammatory responses as a result of immune system recognition or may induce toxic reactions stemming from byproducts produced during material degradation [96]. Furthermore, although certain polymeric materials can be eliminated from the body upon degradation, they may still present a risk of chronic inflammation, particularly in localized environments such as joints [31].

### 4.2. Individual Differences in Efficacy

The pathological mechanisms underlying osteoarthritis are intricate and display considerable interindividual variability, resulting in diverse patient responses to drug delivery systems. While nanoparticles may exhibit substantial joint retention in certain patients, their effectiveness may be markedly diminished in others due to increased synovial fluid flow or the presence of biological barriers [97]. Additionally, the severity of synovitis can influence the distribution and retention of drug delivery, thereby further constraining the consistency of therapeutic efficacy.

### 4.3. Difficulty in Clinical Translation

While laboratory investigations demonstrate that innovative drug delivery systems exhibit considerable efficacy, the process of translating these findings into clinical practice is both intricate and expensive. For instance, the production of gene therapy vectors and biomimetic materials entails complex manufacturing processes that require rigorous purification and quality control measures, thereby posing challenges for large-scale implementation. Maudens et al. [28] have noted that numerous delivery systems presently fail to comply with industrial production standards, resulting in heightened manufacturing expenses and regulatory challenges. Furthermore, the absence of long-term safety data for certain materials in clinical trials further complicates the drug approval process.

### 4.4. Trade-Off Between Efficacy and Dosage

Controversies persist concerning the optimization of dosage in innovative drug delivery systems. Although sustained-release systems are designed to extend the duration of drug efficacy, the challenge of maintaining effective drug concentrations while preventing toxic side effects associated with elevated drug levels remains an unresolved concern [98].

## 5. Outlook, Clinical Translation Potential, and Summary

### 5.1. Outlook

Future research in drug delivery systems is directed towards the development of intelligent, multifunctional, personalized, and sustainable technologies aimed at effectively addressing the treatment challenges associated with OA.

Intelligent drug delivery systems facilitate targeted release of therapeutics at sites of arthritis-related inflammation by responding to specific pathological environmental cues, including pH, temperature, and redox state, thereby enhancing therapeutic efficacy. Lan et al. [99] developed a stimulus-responsive therapeutic platform utilizing nanomicelles that integrate mechanisms responsive to the low pH and elevated activity of MMP-13 characteristic of the inflammatory microenvironment in arthritis. This system enables the precise release of anti-inflammatory agents, such as psoralen, directly into the joint cavity, resulting in significant improvements in both inflammatory conditions and cartilage degeneration associated with osteoarthritis. Furthermore, this intelligent delivery mechanism not only optimizes drug utilization but also minimizes systemic side effects, thereby offering innovative strategies and technical support for precision medicine.

Multifunctional delivery systems integrate anti-inflammatory, antioxidant, and car-tilage regeneration capabilities into a single platform, aiming to achieve synergistic ther-apeutic outcomes through optimized material design. For instance, Yang et al. [100] de-veloped a nanoparticle system that co-delivers anti-inflammatory agents and chondro-genic compounds. This approach offers a dual advantage: enhancing retention within the joint cavity—a critical factor in osteoarthritis treatment—to not only suppress inflamma-tion but also actively promote the regeneration of degraded cartilage. In a more advanced strategy, Nakamura’s team [101] employed a gene-editing approach, utilizing CRISPR/Cas9 to target and disrupt genetic pathways responsible for cartilage breakdown, combined with antioxidants for added cel-lular protection.

Despite their different mechanisms, both approaches exemplify a "multi-targeting" philosophy, creating versatile nanoparticles designed to concurrently combat inflamma-tion and oxidative stress while promoting repair. Furthermore, the primary challenge lies in translating these promising in vitro results to the complex, heterogeneous environment of the human joint, where biological realities could lead to unforeseen complications. Ultimately, these innovative solutions must be rigorously validated against the unpredictable physiology of the human body.

Personalized treatment entails the examination of patients’ biomarkers and genetic information to create targeted drug delivery systems that address the unique therapeutic requirements of individuals with various pathological conditions. Kou et al. [102] highlighted that the development of customized drug delivery platforms, specifically designed to accommodate the distinct inflammatory milieu and cartilage damage profiles of patients with OA, can markedly enhance treatment efficacy.

The advancement of sustainable materials is essential for facilitating cost-effective and environmentally sustainable production processes. Rahimi et al. [103] suggested that the application of green chemistry and biosynthesis technologies can lead to the creation of biodegradable, non-toxic, and stable drug delivery systems, thereby substantially lowering expenses in clinical applications. Additionally, microspheres and hydrogels derived from either natural or synthetic polymers can fulfill biocompatibility standards while concurrently reducing environmental repercussions [104].

### 5.2. Clinical Translation Potential

While the diverse NDDS platforms discussed offer significant therapeutic promise for OA, their readiness for clinical translation and commercial viability varies considerably. A comparative assessment based on efficacy, scalability, biocompatibility, and cost-effectiveness is crucial to identify the most promising candidates.

Liposomes: Demonstrated efficacy in preclinical models for sustained release and targeted delivery of various payloads (anti-inflammatories, genes, antioxidants). PEGylation improves stability and circulation time. However, concerns regarding in vivo stability (degradation by serum proteins/enzymes), complex manufacturing processes leading to batch variability, high production costs (estimated $500–$1000 per dose), and limited drug loading capacity pose significant hurdles to scalability and cost-effectiveness. Clinical translation is nascent, with PEGylated formulations showing promise in early trials but lacking extensive late-stage clinical data.

Polymer Microspheres: Excel in sustained release (weeks to months), beneficial for reducing injection frequency. Materials like PLGA are well-established and biocompatible. Challenges include precise control over degradation kinetics to match drug release profiles, potential inflammatory responses to acidic degradation byproducts of some polymers (e.g., PLA, PLGA), and complexity in achieving deep cartilage penetration due to larger size. Manufacturing scalability is moderate but requires optimization. Clinical adoption is progressing, particularly for corticosteroid delivery (e.g., dexamethasone-loaded PLGA microspheres), but broader application for complex payloads needs more validation.

Nanoparticles (Polymeric, Metallic, Lipid-Based NLCs/SLNs): Offer advantages in cellular penetration, high surface area for functionalization (targeting ligands, stimuli-responsiveness), and versatility in payload delivery (drugs, genes, imaging agents). Surface engineering (e.g., cartilage-targeting peptides like WYRGRL) enhances joint retention and specificity. Concerns revolve around long-term biodistribution, potential systemic toxicity, and biodegradation pathways, especially for inorganic nanoparticles. Scalability of complex engineered nanoparticles can be challenging. Nanostructured Lipid Carriers (NLCs) show promise with better drug loading than SLNs and good biocompatibility. While numerous promising preclinical studies exist (e.g., siRNA delivery, antioxidant enzymes), robust clinical trial data in OA specifically is still emerging. Regulatory pathways for complex nanomedicines require careful navigation.

Hydrogels: Stand out for their excellent biocompatibility, injectability, sustained and localized drug delivery, and ability to provide a protective microenvironment for cells (e.g., stem cells, chondrocytes) and sensitive biologics. They can be engineered for stimuli-responsiveness (pH, enzymes, temperature). Thermosensitive hydrogels are particularly attractive for clinical use. Challenges include ensuring adequate mechanical strength in the joint space, precise control over drug release kinetics, and managing potential inflammatory responses if degradation byproducts accumulate. Injectable thermosensitive hydrogels are the most clinically advanced among NDDS for OA, with formulations progressing to Phase III trials for pain relief. Their relatively simpler composition (often based on natural polymers like HA or synthetic ones like poloxamers) aids regulatory approval and scalability compared to highly complex nanoparticles.

Biomimetic Delivery Systems (Cell membrane-coated NPs, Exosomes, ECM-mimics): Represent a cutting-edge approach leveraging natural targeting and evasion mechanisms. Exosomes, in particular, possess inherent tropism, low immunogenicity, and the ability to carry complex molecular cargo (proteins, nucleic acids). They show high therapeutic potential in preclinical OA models. However, major challenges include standardization of isolation, characterization, and large-scale production, batch-to-batch variability, loading efficiency, regulatory complexities due to their biological nature, and high costs. Despite these hurdles, exosome-based therapies dominate recent intellectual property activity (e.g., 62% of 2023 OA NDDS patents), indicating strong commercial interest. Cell membrane-coated nanoparticles improve circulation and targeting but share similar production and standardization challenges. These systems hold immense long-term promise but face significant translational barriers in the near term.

Based on the current landscape, hydrogel-based systems, particularly injectable thermosensitive formulations, exhibit the most immediate commercial and clinical translation potential for OA. Their favorable biocompatibility profile, established use in other medical applications, clinical progression (Phase III), relative ease of manufacturing scalability, and ability to address key OA treatment needs (sustained release, local delivery, cell support) position them as front-runners. Liposomes and polymer microspheres, especially with continued refinement (e.g., targeting, stability), offer viable pathways, particularly for specific drug classes, but cost and manufacturing challenges need addressing. Nanoparticles show high therapeutic versatility and innovation potential but require more extensive safety and manufacturing data for widespread adoption. Biomimetic systems, especially exosomes, represent the future frontier with high targeting potential but face substantial standardization, regulatory, and cost hurdles before large-scale clinical use becomes feasible.

### 5.3. Summary

Innovative drug delivery systems exhibit significant potential in the management of OA, owing to their advantages in sustained release, targeted delivery, and biocompatibility. These characteristics position them as promising alternatives for OA treatment. Future advancements in these systems are anticipated to focus on personalization, intelligence, and multifunctionality, thereby facilitating the integrated use of gene therapies, antioxidants, stem cells, and growth factors in OA management. Nonetheless, current challenges primarily pertain to issues of biocompatibility, preparation methodologies, control of drug release, and the accumulation of clinical evidence. Subsequent research endeavors should prioritize the optimization of material properties, the simplification of preparation techniques, and the validation of long-term safety and efficacy through clinical trials. Future NDDS must prioritize scalable manufacturing (microfluidic liposome synthesis) and personalization (biomarker-guided hydrogels). CRISPR-loaded exosomes and 4D-printed stimuli-responsive scaffolds represent next-generation frontiers. With continued technological refinement and interdisciplinary collaboration, these drug delivery systems have the potential to address the limitations associated with conventional treatments, ultimately offering more effective and safer therapeutic options for patients with OA and achieving a comprehensive translational application of novel drug delivery systems in clinical practice.

## Figures and Tables

**Table 1:** Application of NDDSs (Novel Drug Delivery Systems).

Delivery System	Drugs	Model	Sustained Release Time	Result
Dex-Lips (Liposome)	Dexamethasone	Mouse (DMM)	>4 day	Promoting the M2 polarization of synovial macrophages alleviated osteoarthritis in miR-204/-211 deficient mice and improved pain symptoms.
Liposomal TGF-β1 (Liposome)	TGF-β1	The dentine-pulp complex	>4 day	Long-term delivery of TGF-β1 and maintaining therapeutic concentrations in the cellular microenvironment promote odontogenic differentiation of human dental pulp stem cells.
AST@Lip-FA (Liposome)	Astaxanthin	Mouse (ACLT)	>1 week	Effectively eliminate the overexpressed ROS and NO in the OA microenvironment, thereby reducing the invasion of synovial macrophages, alleviating related inflammation and tissue damage, and promoting cartilage tissue repair.
siBAK1@Lips (Liposome)	siRNA	Mouse (DMM)	>1 week	Reduce the friction of articular cartilage and release cartilage/osteoclast-targeted siBAK@Lips, thereby inhibiting inflammation and delaying the progression of osteoarthritis through the cGAS-STING pathway.
WY-Lip/UA (Liposome)	Urolithin A	Rat (DMM)	>2 weeks	Effectively target chondrocytes and promote cell uptake, enhance mitochondrial function recovery, reactive oxygen species clearance, and maintain chondrocyte homeostasis by increasing mitochondrial autophagy.
Polyphosphazene (microsphere)	Ibuprofen	—	>3 weeks	It has non-toxic degradation products, can reduce gastrointestinal side effects, features controlled or sustained release properties, improves bioavailability, and reduces dosing frequency.
Poly (lactic-co-glycolic acid) (PLGA) (microspheres)	Dexamethasone	Pre-treatment (IL-1α)	>3 weeks	Enhanced its mechanical function to counteract the harmful effects of local pro-inflammatory cytokine exposure.
gelatin (microspheres)	IL-4 /IL-13	Pre-treatment (IL-1β/LPS)	>2 weeks	Prolong the half-life of anti-inflammatory cytokines while reducing their clearance during periods of low disease activity.
GM-Lipo@ARB (microspheres)	Arbutin (ARB)	Pre-treatment (IL-1β)	>4 weeks	By inhibiting the activation of NF-κB for anti-inflammatory effects and activating the Nrf2 pathway for antioxidant stress, the progression of OA can be alleviated.
siRNA-micelles (microspheres)	siRNA	3D stem cell aggregates	>4 weeks	Enhance the formation, organization, and development of tissues and organs based on cell aggregates, providing new solutions for addressing fundamental biological issues and disease treatment.
F127/COS/KGNDCF (Nanoparticles)	F127/KGN	Pre-treatment (IL-1β)	Controllable	Control the immediate and sustained release of DCF and KGN through the thermal responsiveness of expansion and contraction, as well as the composition ratio of F127 or KGN to COS.
DM (Nanoparticles)	Dopamine	Pre-treatment (IL-1β)	>24 h	By inhibiting the generation of intracellular ROS and RNS, and activating antioxidant enzymes through autophagy, cartilage degradation and the progression of OA can be suppressed.
RB@MPMW (Nanoparticles)	Rapamycin	Rat (ACLT)	Controllable	By activating the AMPK-SIRT1-PGC-1α signaling pathway, they enhance the energy metabolism of chondrocytes, further rescue cell apoptosis in vitro, and inhibit cartilage degeneration in vivo.
PCCGA (Hydrogel)	—	—	Controllable	Exhibits good biocompatibility, antioxidant activity, ROS scavenging ability, and promotes cell migration.
HA-PBA-Gel (Hydrogel)	Phenylboronic acid	Pre-treatment (H_2_O_2_)	Controllable	A convenient and effective solution has been provided for the manufacture of cartilage tissue engineering scaffolds with inherent antioxidant properties.
ECM-mimicking@ADSCs (Hydrogel)	ADSCs	Rat (ACLT/MMx)	>1 week	In the rat OA model induced by surgery, significant relief was observed in cartilage degeneration, joint inflammation, subchondral bone loss, and structural damage.
Fibrin-HA (hydrogel)	Gapmer	Gene knockdown	>2 weeks	Continuously release and effectively suppress the expression of genes related to cartilage degeneration.
HA-EGCG (hydrogel)	EGCG	Rat (DMM)	>1 month	Significantly induce the polarization of synovial macrophages to the M2 phenotype and reduce synovial inflammation, thereby maximizing the role of ADSCs in repairing OA cartilage damage.
NM-LANPs@Ru (cell-membrane)	L-arginine	Rat (injection of papain)	>1 week	Based on the synthesis of neutrophil membranes, the effective regulation of autophagy can inhibit inflammatory C28/I2 cell apoptosis, thereby effectively curing OA.
CAP-GFP-Lamp2b (Exosomes)	microRNA-140	Rat (DMM)	>24 h	By modifying exosomes to avoid the phagocytic action of monocytes on microRNA-140 and the degradation by enzymes in the extracellular matrix, the vitality of OA cartilage can be restored.
dECM-BMSC-Exos (Exosomes)	BMSCs	Mouse (DMM)	>24 h	Enhancing the relief of OA progression by upregulating miR-3473b to improve anabolic metabolism and migration, as well as to inhibit chondrocyte apoptosis.
ERMs@siM13 (Micelles)	siM13	Mouse (DMM)	>24 h	By restoring the balance of cartilage matrix metabolism, the progression of early OA was effectively delayed. In addition, ERMs@siM13 real-time reports on the progress of OA can be provided by promptly responding to different levels of MMP13.
RH-SLNs (SLNs)	RH	Rat (MIA)	>3 weeks	Significantly inhibits inflammatory response, oxidative stress, and cartilage degeneration in arthritis rats, and rapidly penetrate cartilage tissue and are continuously released for 3 weeks in a rat osteoarthritis model.
LRX-NLCs-Gel (NLCs)	LRX	Rat (MIA)	>24 h	Not only improve the pain of rats’ OA, but also reduce the proliferation of synovium, inflammation and other indicators, as well as improve the cartilage structure.

## Data Availability

Data are contained within the article.

## Abbreviations

The following abbreviations are used in this manuscript:

DDS	Drug delivery systems
NDDS	Novel drug delivery systems
MMPs	Matrix metalloproteinases
ADAMTS	A disintegrin and metalloproteinase with thrombospondin motifs
IL-1β	Interleukin-1 beta
TNF-α	Tumor necrosis factor-alpha
ROS	Reactive oxygen species
NF-κB	Nuclear factor kappa-light-chain-enhancer of activated B cells
MAPK	Mitogen-activated protein kinase
